# Our Undertaking

**Published:** 1841-06

**Authors:** 


### THE WESTERN JOURNAL

Vol. III.—No. VI.

LOUISVILLE, JUNE 1, 1841.

---

### OUR UNDERTAKING.

This number completes our third volume. To say that we have made the work what it should have been; or what, under other circumstances than those in which we have been placed, we ourselves might have made it, would be boastful. Nevertheless, we are gratified in being able to state that its patronage has been steadily increasing from the commencement; and has become such, as to guaranty its permanence. Being the only medical or scientific journal in the Valley of the Mississippi and the Lakes, its cognomen "Western" cannot be regarded as presumptuous; and we trust that our Western brethren will think with us, and act accordingly. Of course we are not hinting to them, in the imperative mood, "*subscribe!*" Such hints we leave, gentle reader, to our publishers;—happy to know that we could not confide it in abler hands. But we *are* hinting, that we should be glad to receive *good* communications, from all parts of the aforesaid Western Valley. All communications are either good, bad or indifferent. Heretofore we have not had more bad ones than was supportable, but the number of indifferent has been rather too great, of the good too small. The bad give us no trouble—the indifferent no pleasure—the good no trouble and much pleasure. It is quite natural, then, that we should prefer them to all others; and we assume that our readers have nearly the same preference.

